# Expression and role of RIP140/NRIP1 in chronic lymphocytic leukemia

**DOI:** 10.1186/s13045-015-0116-6

**Published:** 2015-03-04

**Authors:** Marion Lapierre, Audrey Castet-Nicolas, Delphine Gitenay, Stéphan Jalaguier, Catherine Teyssier, Caroline Bret, Guillaume Cartron, Jérôme Moreaux, Vincent Cavaillès

**Affiliations:** IRCM, Institut de Recherche en Cancérologie de Montpellier, Montpellier, F-34298 France; INSERM, U1194, Montpellier, F-34298 France; Université de Montpellier, Montpellier, F-34298 France; Institut régional du Cancer de Montpellier, Montpellier, F-34298 France; Pharmacie, CHU Montpellier, Montpellier, F-34298 France; Département d’Hématologie Biologique, CHU Montpellier, Montpellier, F-34298 France; Institut de Génétique Humaine, CNRS UPR 1142, Montpellier, F-34298 France; Département d’Hématologie Clinique, CHU Montpellier, Montpellier, F-34298 France; UMR-CNRS 5235, Montpellier, F-34298 France

**Keywords:** Chronic lymphocytic leukemia, Cell signaling, RIP140/NRIP1, Prognosis marker

## Abstract

RIP140 is a transcriptional coregulator, (also known as NRIP1), which finely tunes the activity of various transcription factors and plays very important physiological roles. Noticeably, the RIP140 gene has been implicated in the control of energy expenditure, behavior, cognition, mammary gland development and intestinal homeostasis. RIP140 is also involved in the regulation of various oncogenic signaling pathways and participates in the development and progression of solid tumors. During the past years, several papers have reported evidences linking RIP140 to hematologic malignancies. Among them, two recent studies with correlative data suggested that gene expression signatures including RIP140 can predict survival in chronic lymphocytic leukemia (CLL). This review aims to summarize the literature dealing with the expression of RIP140 in CLL and to explore the potential impact of this factor on transcription pathways which play key roles in this pathology.

## Introduction

### Chronic lymphocytic leukemia

Chronic lymphocytic leukemia (CLL) is the most common form of leukemia in Western countries and mainly affects elderly individuals. CLL is characterized by the accumulation of malignant mature B cells in bone marrow, blood and lymphoid tissues. The clinical course of CLL is extremely heterogeneous, with many patients presenting an indolent disease, whereas others exhibit an aggressive pathology and require treatment [1,2].

The diagnosis of CLL is based on biological criteria including the presence of a chronic lymphocytosis (≥5.10^9^/L) with a typical phenotype characterized by a κ or λ light chain restriction, the co-expression of B cell markers (CD19, CD20, CD22 with a low density, CD23) with the CD5 antigen (in the absence of other pan-T cell markers) and the expression of additional markers like CD200 or CD43 [3]. These characteristics are also sufficient for the distinction between CLL and other mature B cell disorders such as prolymphocytic leukemia, hairy-cell leukemia, mantle-cell lymphoma, or other lymphomas that can mimic CLL.

CLL has previously been considered as a single entity with a variable clinical course. Recently, there has been considerable progress in the identification of molecular and cellular markers that may predict disease progression in patients with CLL [2]. Particularly, mutational profiles of *Ig* genes and cytogenetic abnormalities have been demonstrated to display a strong prognostic value (see below).

### The transcription factor RIP140

**Figure 1:**
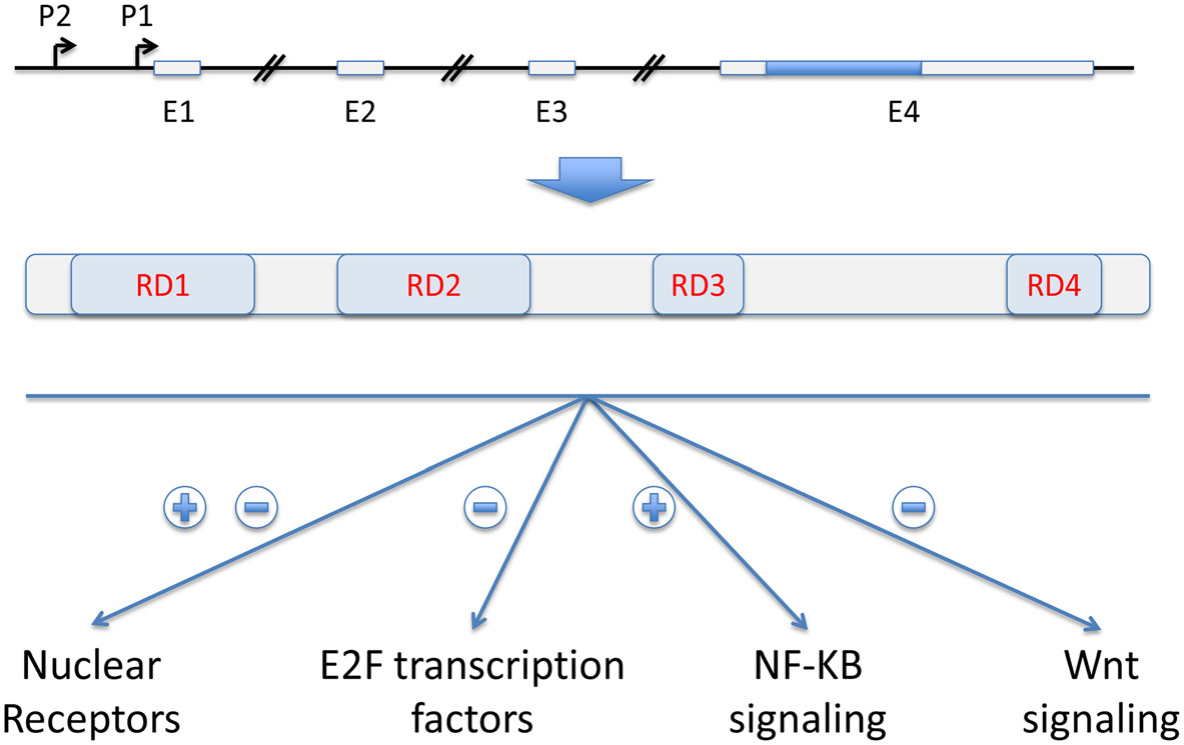
**Structure of the RIP140 gene and protein.** Schematic representations of the RIP140 gene and protein (not scaled). (Top panel) The two promoters are shown together with the four exons (E1 to E4) which are represented by small rectangles, the blue one corresponding to the RIP140 coding sequence. (Middle panel) The box represents the RIP140 molecule showing the four different repressive domains (RD). (Bottom panel) The different nuclear signaling pathways either inhibited (−) or stimulated (+) by RIP140 are indicated.

The transcription cofactor RIP140 (receptor-interacting protein of 140 kDa), known as nuclear receptor-interacting protein 1 (NRIP1), was first identified in human breast cancer cells through its interaction with the estrogen receptor α [4]. RIP140 was also shown to interact with many other nuclear receptors and transcription factors (for a review see [5]). RIP140 mainly acts as a transcriptional repressor by means of four inhibitory domains (see Figure 1) that recruit histone deacetylases or C-terminal binding proteins [6,7]. Moreover, several post-translational modifications, such as sumoylation and acetylation, play important roles in controlling the subcellular location and repressive activity of RIP140 (for a review [8]). *RIP140* is an ubiquitously expressed gene, located on chromosome 21 in humans, whose transcription is finely regulated at the transcriptional level [9,10]. The *RIP140* gene exhibits two promoters and several exons, the last one encompassing the whole coding sequence (see Figure 1).

The physiological importance of RIP140 has been evaluated using mice devoid of the *RIP140* gene (RIPKO mice). These animals are viable but display a wide range of phenotypic alterations in various tissues and organs, such as infertility of female mice [11] or reduced body fat content [12]. A more recent work demonstrated that they suffer severe cognitive impairments [13]. Besides these important physiological roles, RIP140 has recently been shown to regulate key oncogenic signaling pathways that impact cancer initiation and progression [14-16].

### RIP140 and hematopoietic stem cells

Hematopoietic stem cells (HSCs) are rare and multipotent, self-renewing precursor cells which are able to generate all specialized cells of the blood system [17]. A precise regulation of HSC proliferation and cell fate decisions is necessary to maintain ongoing production of mature blood cells throughout adult life and for rapid, regenerative responses to hematologic injury. Several studies indicated the importance of active maintenance of HSC stem cell function and identified genes that perturb HSC quiescence and disrupt stem cell maintenance and homeostatic blood cell production [18,19]. Many of these genes encode transcription factors or cell cycle regulators that directly modulate the proliferative activity of HSC.

By using gene expression microarray and systems biology tools, a functional network reconstruction was performed in cord blood CD133+ HSCs in order to identify genes involved in stemness [20]. The *RIP140* gene was found highly expressed in HSC, as well as in mesenchymal and neural stem cells. Another study identified the *RIP140* gene as being downregulated in mobilized HSC compared to HSC at steady-state [21]. The same report described a decrease in RIP140 expression in leukemic HSC obtained from the bone marrow of Jun B-deficient mice (a model of chronic myelogenous leukemia) as compared to HSC from wild-type mice. Altogether, these data suggested that RIP140 might be an important factor required for the maintenance and function of normal quiescent HSC. Concerning its expression in the different hematopoietic cellular types, a study reported a low *RIP140* gene expression in T cells and the highest expression level in NK cells [22] (see Table 1).

**Table 1:** **Expression of the** ***RIP140*** **gene in the different hematopoietic cellular types**

**Cell type**	**T cells**	**B cells**	**Monocytes**	**NK cells**	**Granulocytes**
RIP140 relative expression	100	576	453	1,825	138

The expression levels of the *RIP140* gene were determined in normal granulocytes and FACS-sorted monocytes, B cells, T cells and NK cells. Values are expressed as percent of levels measured in T cells.

(Adapted from [22]).

### RIP140 as a prognostic marker in CLL

Genetic aberrations such as recurrent losses or gains of chromosomal material as well as mutations of key tumor suppressors have been identified in CLL. Approximately 80% of CLL cases exhibit aberrations in a few recurrently affected chromosomal regions. These aberrations are important “drivers” of the disease and are also considered as prognosis biomarkers. CLL has turned out to be a multifaceted disease with pathogenic mechanisms including genetic aberrations, antigen processing, and microenvironmental interactions. As a consequence, there is a remarkable heterogeneity in the clinical course among patient subgroups with distinct genetic features.

According to the mutation status of immunoglobulin heavy chain variable gene segments (IGHVs), CLL patients can be classified in two groups displaying a very different clinical course [23]. Patients with an unmutated *IGHV* genetic profile show an unfavorable evolution, whereas patients with mutated *IGHV* have a better prognosis. Other poor prognostic markers in CLL are the chromosomal deletion at 11q (TP53 locus) or 17p (ATM locus). Concerning patients with del(11q), the poor outcome of is overcome by chemoimmunotherapy with fludarabine, cyclophosphamide, rituximab (FCR) [2]. More recently, recurrent mutations of NOTCH1, SF3B1, and TP53 have been shown to be associated with an adverse prognostic impact in CLL [24]. With the development of microarray technology and transcriptomic analyses, additional markers such as zeta-associated protein 70 (ZAP70), lipoprotein lipase (LPL), CLL upregulated 1 (CLLU1), transcription factor 7 (TCF7), T cell leukemias/lymphoma (TCL1A), or a disintegrin and metalloprotease domain 29 (ADAM29) have been characterized [25,26].

**Figure 2:**
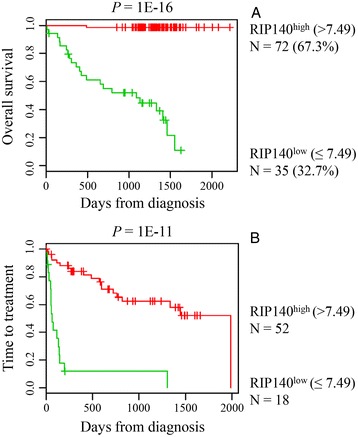
**Prognostic value of RIP140 in CLL patients. (A)** Patients of the Herold’s cohort (*n* = 107) were ranked according to increasing RIP140 expression and a maximum difference in OS was obtained with an expression cutoff of 7.49 using the Maxstat R function. **(B)** Low RIP140 expression is associated with a shorter time to the first treatment in CLL patients. The data were normalized using the robust multichip average (RMA) method and Affymetrix gene expression data are publicly available via the online Gene Expression Omnibus (http://www.ncbi.nlm.nih.gov/geo/) under accession number GSE22762, and GSE25571.

RIP140 was first identified as a CLL prognostic factor in a gene expression-based study using a cohort of 130 patients [22]. Furthermore, the *RIP140* gene was shown as being part of a recently reported eight-gene expression signature which defined a risk score for CLL patients [27]. Low expression of RIP140 is associated with poor prognostic value for overall survival (OS) and time to treatment requirement (Figure 2). Although no studies have exclusively been focused on RIP140 in CLL, several other published data reported the deregulation of RIP140 gene expression in this pathology (Table 2). Many of these studies showed its differential expression with regard to *IGHV* mutational status [28-30]. In the study of van’t Veer et al., it was observed that deletion 17p13, associated with short treatment-free intervals, was more frequent in RIP140 negative cases. Similarly, deletion 11q22, which is accepted as an indicator of unfavorable prognosis [31], was only seen in RIP140 negative cases [22]. More recently, some of us reported a new gene expression-based risk score in CLL specimens corresponding to 20 genes (22 probe sets), whose expression splits patients of two independent cohorts into two risk categories [32]. Interestingly, the RIP140 expression kept prognostic value in multivariate COX analysis leading to a new risk stratification of patients with CLL.

**Table 2:** **Main studies describing the deregulation of the** ***RIP140*** **gene expression in CLL**

**Reference**	**Date**	**Number of CLL patients**	**Association with VH mutation**	**Correlation with survival**
Haslinger et al. [28]	2004	100	Yes	–
Vasconcelos et al. [29]	2005	145	Yes	–
Oppezzo et al. [30]	2005	127	Yes	–
Van’t veer et al. [22]	2006	130	NS	NS
Herold et al. [27]	2011	151	Yes	Yes
Samra et al. [32]	2014	107	Yes	Yes

NS not significant.

Finally, emerging evidence indicates that the stromal tumor microenvironment plays important roles in the pathogenesis and progression of CLL. The expression of stromal-associated genes has been evaluated using gene expression profiling [33]. Interestingly, the *NRIP1* gene was found to be underexpressed in cells isolated from peripheral blood, bone marrow, and lymph nodes from CLL patients in comparison with healthy donors.

### Effect of RIP140 on key signaling pathways in CLL

Since its identification as an estrogen receptor binding protein [4], RIP140 has been characterized as a partner for many transcription factors involved in major oncogenic signaling pathways (Figure 1). Although no published studies support that RIP140 alters the biology of CLL, some of the pathways targeted by RIP140 are highly relevant in CLL biology.

#### Effect on NF-κB signaling

In CLL, NF-κB has been found to be activated regardless of the disease stage or treatment status [34] and to confer survival benefit through induction of anti-apoptotic proteins including XIAP, BCL-XL, and FLIP [35]. In particular, RelA binding complexes have been demonstrated to be constitutively active in peripheral blood samples of CLL patients, their activation being dependent on the action of the transcription activator signal transducer and activator of transcription-3 (STAT3) [36]. More recently, a role for RelB and RelA has been demonstrated by studying B cells isolated from bone marrow aspirates of CLL patients. RelB activity appeared not only to sustain tumor cell survival but also to enhance cell sensitivity to proteasome inhibitor [37]. More generally, the canonical and non-canonical NF-κB pathways seem to cooperate to CLL progression.

Noticeably, Zschiedrich et al. have reported that RIP140 establishes direct protein-protein interactions with the NF-κB subunit RelA and functions as a coactivator for proinflammatory cytokine gene promoter transcription in macrophages [38]. This coactivator function of RIP140 for NF-κB activity relies on the cooperation with histone acetylase cAMP-responsive element binding protein (CREB)-binding protein (CBP). Treatment of macrophages by TLR ligands such as LPS increased Syk-mediated tyrosine phosphorylation of RIP140 and its interaction with RelA. This also induced the recruitment of the E3 ligase SCF to Syk-phosphorylated RIP140, thus conducting to the degradation of RIP140 and to inactivation of genes encoding inflammatory cytokines [39].

#### Regulation of Wnt signaling

The Wnt signaling pathway plays a crucial role in the specification and development of hematopoietic stem cells and their microenvironment [40]. There is a growing body of evidence that Wnt signaling, known to play a critical role in various types of cancer, also exerts a key function in B lymphoid malignancies, particularly in CLL [41]. Wnt proteins are overexpressed in primary CLL cells and several physiological inhibitors are partly inactivated in this pathology [42]. Furthermore, the transcription factor lymphoid enhancer binding factor-1 (LEF-1) is highly overexpressed in CLL cells, as compared to normal B cells [43]. Moreover, LEF-1 controls several genes relevant in CLL biology and several components of the Wnt signaling pathway significantly influence CLL cell survival. Salinomycin treatment was shown to inhibit Wnt signaling and induce apoptosis of CLL cells [44]. Nitric oxide-donating acetylsalicylic acid (known to present antitumor effect in Wnt active cancers) induced apoptosis of primary CLL cells and reduced significantly tumor growth in a CLL xenograft murine model [45].

Interestingly, our laboratory has recently reported that RIP140 was a key regulator of the Wnt signaling pathway in mouse and human intestinal epithelial cells [16]. RIP140 increases the transcription of the *APC* gene promoter, the major tumor suppressor gene in colon cancer. As a consequence, RIP140 inhibits β-catenin activation, resulting in decreased expression of Wnt target genes including c-Myc, c-Jun, endothelin-1, or jagged-1. In CLL, endothelin-1 was demonstrated to promote survival and chemoresistance through endothelin receptor A [46].

#### Effect on other signaling pathways

RIP140 regulates other signaling pathways highly relevant for CLL biology. Indeed, RIP140 has been identified and characterized as a major partner for nuclear receptors [4,5] which have been shown to be key players in CLL. For instance, high-dose glucocorticoids are used in the treatment of CLL patients [47] and the nuclear receptor PPARα seems to be involved in the resistance to glucocorticoid-mediated cytotoxicity [48]. Another study reported that B-CLL cell survival/viability was decreased as a result of LXR agonist treatment [49]. In addition, the majority of patients with CLL exhibit a significant expression of ERβ, suggesting that this nuclear receptor might be relevant in CLL and used as therapeutic target [50]. Obviously, other signaling pathways important for CLL biology such as p53, Notch, or Hedgehog [51] could be also controlled by RIP140 and further work is needed to uncover and decipher these putative regulatory activities.

### Expression of RIP140 in other types of leukemia

Few studies have analyzed the deregulation of the *RIP140* gene expression in other hematological diseases. RIP140 expression has been found to be significantly upregulated in acute myeloid leukemia (AML) with complex karyotypes and abnormal chromosome 21 [52]. By contrast, RIP140 levels are decreased in acute promyelocytic leukemia (APL), a subtype of AML most commonly characterized by the fusion of the retinoic acid receptor α gene to the promyelocytic leukemia (PML) gene [53,54].

The *NRIP1* gene has also been involved in genomic translocations. Array comparative genomic hybridization analysis performed on a patient diagnosed with a precursor B cell acute lymphocytic leukemia (ALL) with the t(9;22) translocation, identified the *NRIP1* gene as being interrupted at the breakpoints of 21q21.1, and joined with the UHRF1 gene at 19p13.3 as a possible fusion gene, 5′-NRIP1/UHRF1-3′ on the derivative chromosome 19 [55]. Another paper described a breakpoint of t(3;21) (q26;q11) that was assigned to be within the *EVI1* and *NRIP1* gene and generate a putative NRIP1-EVI1 fusion protein [56]. Finally, another fusion event involving the *NRIP1* gene was reported with the open reading frame C21orf34 (also at 21q21 approximately 1 MB apart) in a patient with chronic myelomonocytic leukemia [57]. The fusion took place just upstream of miR-125b-2 and was validated by capillary sequencing. Altogether, these data suggest that the *NRIP1* gene could be involved in the pathogenesis of different types of leukemia.

### Conclusion and future directions

Data clearly suggest that a high expression of RIP140 is a favorable prognostic marker in CLL. However, further work is needed to demonstrate that RIP140 alters CLL biology and to define precisely which signaling pathways are critically regulated by this transcription factor and account for its prognostic value. Several nuclear signaling pathways, including Wnt and NF-κB, are known to be regulated by RIP140 and could be good candidates. In addition, cell models for CLL [58,59] and other types of leukemia [60] have been established and will be useful to define the role of RIP140 at the cellular level. Finally, the use of mouse models with loss and gain of function will be valuable to decipher the role of this gene in normal and tumoral HSC biology.
